# Mixture of Organophosphates Chronic Exposure and Pancreatic Dysregulations in Two Different Population Samples

**DOI:** 10.3389/fpubh.2020.534902

**Published:** 2020-10-28

**Authors:** Mbah Ntepe Leonel Javeres, Saqlain Raza, Ngondi Judith, Fozia Anwar, Rabia Habib, Sajida Batool, Syed Muhammed Nurulain

**Affiliations:** ^1^Department of Biosciences, COMSATS University Islamabad, Islamabad, Pakistan; ^2^Department of Mathematics, COMSATS University Islamabad, Islamabad, Pakistan; ^3^Department of Biochemistry, Yaoundé I University, Yaoundé, Cameroon; ^4^Department of Health Informatic, COMSATS University Islamabad, Islamabad, Pakistan

**Keywords:** diabetes, serum lipase, pesticides, organophosphates, exocrine pancreas

## Abstract

Organophosphates (OP) are a major agrochemical. The application of OP pesticides is expected to increase multifold in the coming decades. The etiology of diabetic diseases is attributed to multiple factors including OP pesticide exposure. The present study investigates pancreatic dysregulation with respect to exocrine enzymes and diabesity in groups of Pakistani and Cameroonian people exposed to a mixture of OP pesticides. Nine hundred and four OP exposed individuals were enrolled for this cross-sectional study after due consent and approval from an ethical review committee. Pesticides' residues were measured by GC-MS spectrometry. Cholinergic enzymes were measured by Elman's method. Serum glucose, insulin, serum amylase, lipase, and triglyceride were measured by spectrophotometry and ELISA; HOMA-IR was determined in OP exposed and non-exposed participants. Stata 15 and R 3.2.0 software were used for statistical analysis of the data. Malathion, chlorpyrifos, and parathion residues were evident in plasma samples. RBC-acetylcholinesterase was significantly depressed in OP exposed groups. In both population samples, investigated pancreatic functions were found to be statistically significantly more dysregulated than non-exposed. OP exposure indicated risk of diabetes and insulin, glycaemia, adiponectin, triglycerides, and TNF-α dysregulations. The study concludes that both OP exposed population groups exhibited a mixture of OP residues and pancreatic dysregulation, although the effect was more pronounced in the Cameroonian population. In addition, serum lipase has a positive correlation with OP exposure and diabetes and may be suggested as an alternate/additional diagnostic marker for diabesity under OP exposure. However, screening of other environmental co-factors with OP for pancreatic dysregulation is suggested.

## Introduction

Organophosphates (OP) are heavily used in agriculture. Their use has been predicted to increase multifold in the coming decades. Organophosphates' primary toxic action is to inhibit, acetylcholinesterase (AChE). Globally, most of the population, particularly in agriculture growing areas, are exposed to OP. Peoples of all ages are exposed to toxicants like OP. Worldwide, organophosphates account for about forty percent of total pesticides use and Pakistan ranks second in the use of pesticides in South Asian countries (1). According to Pouokam et al. (2), about 600 different types of pesticides are registered for use in Cameroon and, among OP, glyphosate and chlorpyrifos are the most commonly used pesticides.

Accumulating evidence shows that there are many secondary toxic manifestation of OP, such as endocrine disruption (3), cardiotoxicity (4), neurobehavioral effects (5), and metabolic effects (6). If we look at endocrine related diseases, type-2 diabetes is increasing steadily around the world, reaching unprecedented rates. In the early 2000s, WHO predicted that around 300 million people would develop type-2 diabetes by 2030 (7). In 2013, the same organization reported the existence of 347 million diabetics worldwide, of which more than 90% had type-2 diabetes and, more recently, the International Diabetes Federation (IDF) reported an overall prevalence of 8.3% which corresponds to 382 million people. About 7.9% of the population suffers from diabetes in Pakistan (8) and it is expected to rank fourth in the world for the burden of diabetic diseases by 2030 (9). Diabetes is also one of the most prevalent disorders in the African sub-continent and it is projected to increase to 7.1% by 2030 (10). The reasons for this rapid increase remain complex and, for the moment, without a definite answer (11).

Several risk factors have been investigated to understand the multifactorial etiology of diabetes, including nutritional, genetic, and environmental factors (12). A number of clinical studies/case reports and other experimental models have provided evidence that shows hyperglycemia is due to OP intoxication (13–17). Kuehn (18) hypothesized that incidence of diabetes surge in relation to days of exposure on agriculture farms. Montgomery et al. (19) identified a number of OP pesticides which may cause diabetes on exposure. It is noteworthy that there are more than 150 different types of OP insecticides which differ in structure and function. Though all OPs have the same mechanism of toxic action, i.e., the inhibition of AChE, toxic manifestation may vary. For instance, Nurulain et al. (20) reported that paraoxon does not cause diabetes or exaggerate diabetes in rats when sub-chronically exposed to them for 2 months. Diabetes has a complex pathology involving hyperglycemia, insulin-resistance, low-grade inflammation, accelerated atherosclerosis, and nephropathies (21–23). The impaired pancreatic exocrine functions for insulin dependent or non-dependent diabetes and OP exposure are not yet fully understood and remain controversial. However, some of the proposed mechanisms are oxidative and nitrosative stress, dysregulation in inflammatory cytokines, paraoxonases enzyme, and metabolism of the liver and stimulation of the adrenal glands (24). Pancreatic dysregulation due to OP intoxication is not only limited to diabetes and insulin resistance; a good number of studies and case reports have shown pancreatitis linked to acute OP intoxication (25–27).

Moreover, low pancreatic insulin was reported in insulin-dependent diabetics by Junglee et al. (28) and Škrha et al. (29). They found significantly low serum lipase levels in juvenile onset diabetics in comparison to healthy non-diabetic human subjects. Rubba et al. (30) found low lipoprotein lipase activity in young ketotic diabetic patients. Nair (31) reported elevated lipase activity in only 29% of the diabetic- ketoacidosis subjects. Vantyghem et al. (25) concluded that pancreatic enzymes like lipase increase with the degree of diabetic disequilibrium and may correlate with metabolic factors like hyperglycemia. Several other studies have reported high activity of serum lipase during type-2 diabetes (25, 32–35). The primary function of lipase is to catalyze the breakdown of triglycerides and is produced by acinar cells of the pancreas. According to Hayden et al. (36), acinar cells may affect the working of endocrine insulin secreting cells through cytokines and growth factors. Lipase has been also reported in the gastrointestinal tract, liver, heart, lungs, and leukocytes (37). Elevated lipase levels indicate the possibility of pancreatitis (38). Chronic pancreatitis exhibits an odd kind of diabetes, termed as type III diabetes, which is different from type I and II in the context that islets cells are damaged by inflammation and fibrotic injury (39). Type III diabetes is little described in scientific literature and has been linked with Alzheimer's disease (40). However, a mild surge in lipase may be noted in a number of pathological conditions, like peptic ulcers and hepatobiliary diseases (37). Lipase is the product of the exocrine part of the pancreas. Pancreatic exocrine inefficiency (PEI) has been reported to be associated with both type I and type II diabetes. PEI has been reported in 25–74% of type I diabetic and 28–54% of type II diabetic patients (39). Pancreatitis and its effect on lipases due to OP exposure has been mainly reported under acute OP poisoning, though OP intoxication occurs both through chronic and acute exposure (41–43). In 1991, Kandalaft et al. demonstrated that exposure to OPs increased the sensitivity of the human exocrine pancreas to acetylcholine (26). Hou et al. (27) also demonstrated that massive OP exposure could lead a total necrosis of the pancreas. It is also evident that humans may be exposed to a mixture of two or more OPs at lower non-lethal doses (44). When two or more OPs are present together at low doses, there might be a cumulative toxicological effect. In 1957, Frawley et al. (45) reported the marked potentiation of malathion toxicity by EPN, when both present together at a non-lethal dose.

The present work was designed to investigate the pancreatic dysregulations caused by chronic organophosphates mixture exposure. The mixture contained two or more than two structurally/functionally different types of OPs that were found in the biological samples at time under exposure. Risk of diabetes with the mixture of OP exposure under environmental conditions will be evaluated. The outcome of the study will provide further rationale for making health policies in public health sectors of the region. To the best of our knowledge, chronic exposure to OPs under natural environmental conditions and pancreatic dysregulation is little reported.

## Materials and Methods

### Study Population and Blood Collection

Subjects from an intensive agricultural area of Pakistan and Cameroon were asked to participate in the study based on their potential exposure to pesticides. It was a cross-sectional study. The study participants were recruited from Mora, Figuil, Njobe, and Sa'a in Cameroon and from Depalpur and Multan in Pakistan. These are cotton growing agriculture areas where organophosphates are mainly applied. Ethical approval was obtained from the ethical review board of COMSATS University Islamabad, Pakistan (CIIT/BIO/ERB/19/90) and Cameroon National Ethical Committee (#488/CE/CNERSH). The study conforms to tenets of the Helsinki Declaration for human subjects in experiments. All the study participants were informed about the purpose of the study and written consent was taken prior to sample collection. The participants were in the age group of 16–60 years, had lived in the OP-sprayed agriculture areas for at least 6 months, and were exposed to pesticides directly or indirectly.

**Figure 1:**
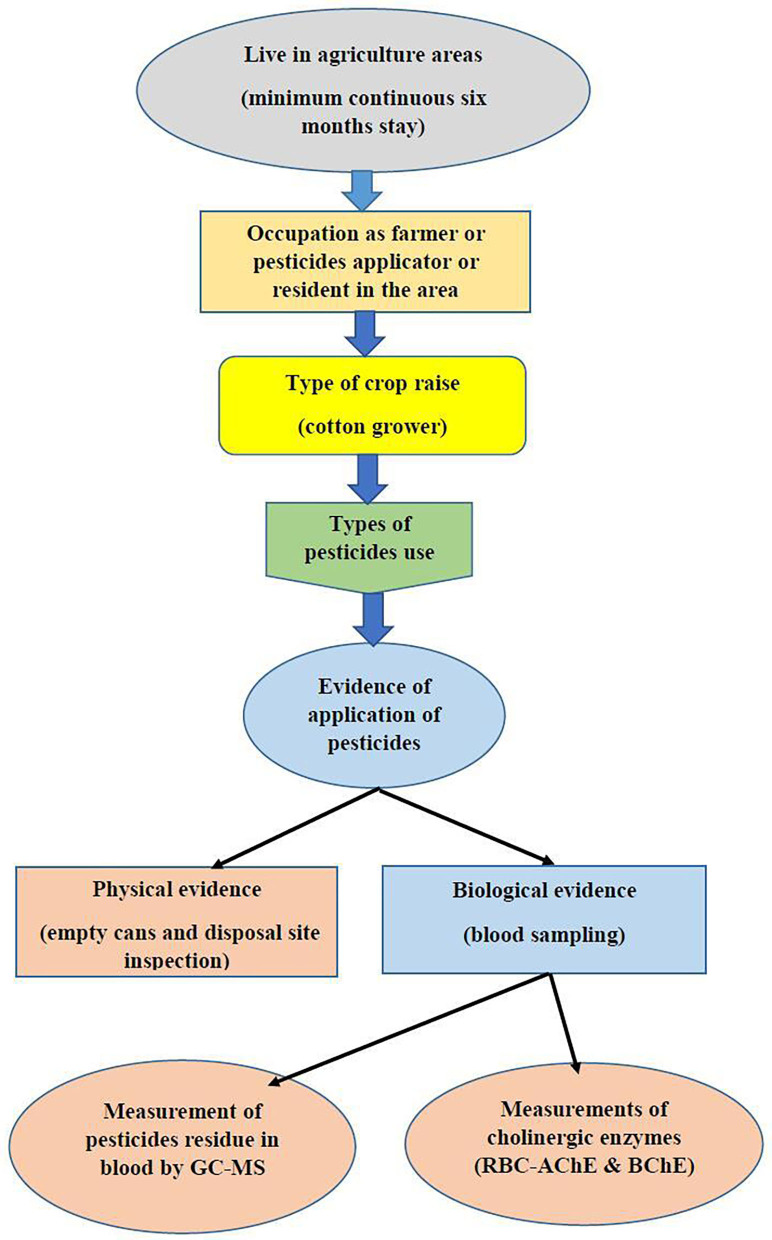
Steps to assess pesticide exposure in the study groups.

Subjects that disclosed use of other OPs, subjects with diabetes, neurological disorders, liver dysfunctions, cancer, or any other type of chronic condition, or subjects from whom GC MS results revealed the presence of other pesticide residue were excluded from the study. Since all the inhabitants in understudied agricultures areas are under direct or indirect exposure to pesticides, unexposed samples were collected from non-agriculture areas with less risk of exposure to pesticides. Figure 1 shows the basic plan for the assesment of exposure in the area.

Questionnaires were provided to collect demographic characteristics and confounding factors such as age, gender, height, weight, tobacco use, work practice characteristics, exposure history, job activities, use of protective equipment, length of time doing present work activity, and proximity of their home to agricultural fields. Our target after power analysis was to collect the blood of 300 exposed participants in Pakistan and same number in Cameroon. The sample size was calculated using the Lorenz formula for cross-sectional studies (46). According to pancreatic disease prevalence in the respective populations (7.9% in Pakistan and 7.1% in sub-Saharan Africa 8,10), the minimum size for each site required to have enough power (with 10% increase to keep the power in case of selection bias) was 124 and 113 for Pakistan and Cameroon, respectively. In Cameroon 125 samples (75 exposed and 50 unexposed) were collected from each site (Figuil, Mora, Sa'a and Njobe). In Pakistan 202 samples (146 exposed and 56 unexposed) were collected from each site (Multan and Delpapur). After screening and exclusion, a total of 904 participants were selected to participate in the study where 300 were exposed and 200 were unexposed from Cameroon and 292 were exposed and 112 unexposed from Pakistan.

**Figure 2:**
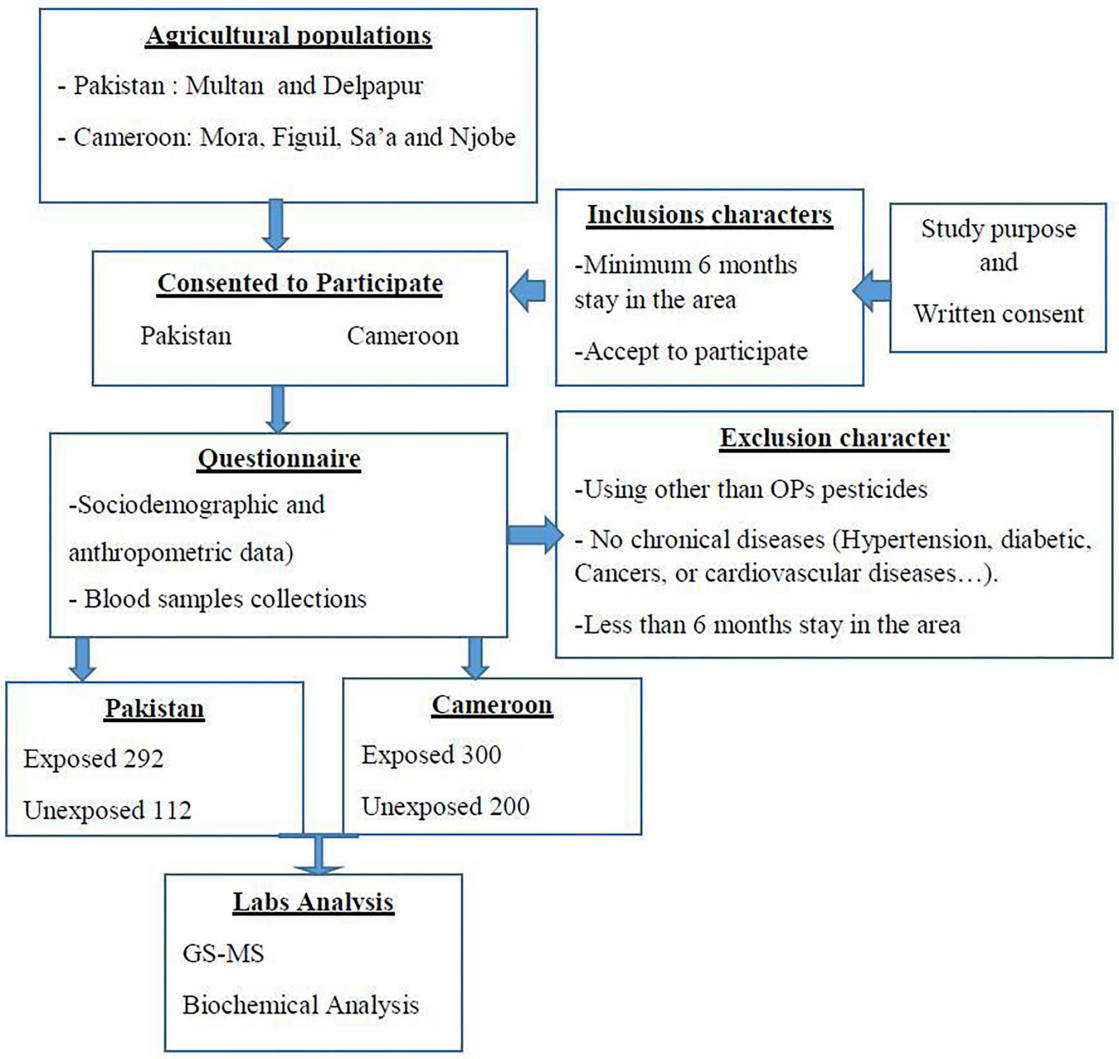
Schematic representation of sampling.

The sampling method was non-probabilistic. Blood samples were drwan once from all participants using aseptic venipuncture and transported to the lab on ice. The collected blood was divided into two tubes for subsequent analysis for plasma and serum fractions, using EDTA-coated and plain glass tubes, respectively. The blood was centifuged at 3,500 rpm for 10 min at 25°C. Plasma and serum thus obtained was then stored at −80°C till further analysis. The samples were analyzed at the IMPM laboratory, Yaounde, Cameroon and the Department of Biosciences laboratories at COMSATS University Islamabad, Pakistan. All those steps are summarized in Figure 2.

### Determination of Pesticide Residues

OP pesticides (chlorpyrifos, malaoxon, and parathion), and other pesticides, such as DDT and bifenthrin, were screened in plasma by gas chromatography coupled with high resolution mass spectrometry (GC-MS System 5975C Agilent, UK) as described earlier (47). The type of pesticides to analyze were chosen on the basis of data collected from farmers regarding the quality of the pesticides used in the fields (Cotton), the pesticides in stock, and after the inspection of the fields and dumping points of abandoned pesticide packages in villages. The column was calibrated at 120°C for 1 min, then set to increase its temperature up to 290°C. The temperatures of the injection orifice and the interface were stabilized at 250°C and the temperature of the detector at 290°C. The injection mode without division and helium with a flow rate of 1.0 ml/min were used as carrier gas. Calibration assay and internal quality control were performed with the addition of known concentrations of commercial solutions of chlorpyrifos, malaoxon, parathion, and internal standards (azobenzene) to drug-free whole blood. The six concentrations used for calibration curves of chlorpyrifos were 0.15, 0.5, 1.0, 1.25, 2.5, and 5.0 mg/L. In case of malaoxon and parathion, these were 0.17, 0.5, 1.0, 1.25, 2.5, 5.00 mg/L and 0.13, 0.5, 1.0, 1.25, 2.5, 5.00 mg/L, respectively. The concentration of internal standard was 0.25 mg/L. Electron impact (EI) mass spectra of pesticides and internal standard were recorded by total ion monitoring. For quantification, the surface/peak ratios of the target ion of different insecticides and azobenzene (m/z 182) were calculated as a function of the concentration of the substance. The data was recorded only when the peak was clearly visible, and the signal-to-noise ratio was greater than three (48). Limit of detection (LOD) and limit of quantification (LOQ) were estimated for the validation of this method. LOD and LOQ were determined by analyzing drug-free blood samples fortified with known drug concentrations. Each concentration was measured in five replicates. LOD was defined as the lowest concentration giving a response of three times the average baseline noise defined from five unfortified samples. LOQ was defined as the lowest observed concentration within 10% of the theoretical concentration with acceptable qualifier ion ratios for all five replicates. 10 blank blood samples were analyzed for chromatographic interference with each analyte.

### Cholinesterase Activity

Organophosphates' exposure was also assessed by measuring cholinesterases [RBC-acetylcholinesterase (RBC-AChE) and butyrylcholinesterase (BChE)]. RBC-AChE and BChE activity were determined using Ellamn's method modified by Worek et al. (49) using Specord 50 plus spectrophotometer (Number; 233H1280C, Analytic Jena, Germany). RBC-AChE was measured from whole blood and plasma was taken for BChE. Blood and plasma dilutions were prepared according to the method described by Worek et al. (38). Spectrophotometric measurement was taken at 436 nm (ε = 10.6 × 10^3^ M^−1^ cm^−1)^ at 37°C for AChE and BChE. For hemoglobin, absorption was noted at 546 nm (ε = 10.8 × 10^3^ M^−1^ cm^−1)^.

### Biochemical Analysis

Lipase was assayed by Biolabo Kit 99891 (kinetic) from BIOLABO SA, Les Hautes Rives, 02160, Maizy France, based on the enzymatic method described by IMAMURA (50). Lipase acts on 1,2-diglyceride to form 2-monoglyceride hydrolyzed to glycerol and free fatty acids by monoglyceride lipase. Glycerol kinase converts glycerol to glycerol-3-phosphate which then generates hydrogen peroxide under the action of glycerol-3-phosphate oxidase. Under the action of peroxidase, H_2_O_2_, 4-AAP (4 Amino antipyrine) participates in the formation of a quinoneimetic complex. The formation rate of this complex was directly proportional to the lipase activity in the specimen and measured at 550 nm. Amylase was assayed by Biolabo Kit 99523 from BIOLABO SA, Les Hautes Rives, 02160, Maizy, France, based on the enzymatic method described by WINN-DEEN (51). Amylase catalyzes the reaction of degradation of 2-chloro-4-nitrophenyl maltotrioside (CNPG_3_) for chloro-nitro-phenol (CNP). The CNP formation rate was directly proportional to the α-amylase activity in the specimen and is measured at 405 nm. Plasma triglycerides were assayed by the enzymatic method using Biolabo Kit 87319 (colorimetric) from BIOLABO SA, Les Hautes Rives, 02160, Maizy, France. Triglycerides (TG) were assayed after enzymatic hydrolysis by lipases to form glycerol and free fatty acids. The indicator was a quinonimine formed from hydrogen peroxide, 4-amino antipyrine, and 4-chlorophenol under the catalytic action of peroxidase. The concentration of quinonimine was proportional to the total concentration of plasma triglycerides at 505 nm (52). Plasma glucose was determined by Biolabo Kit 80009 (colorimetric) from BIOLABO SA, Les Hautes Rives, 02160, Maizy, France. Lipase, Amylase, TG, and glucose wavelengths have been determined using Kenza spectrophotometer (Number; 450 TX), Biolabo, France.

Insulin was determined by the ELISA assay of IBL international (number; RE53171), D-22335 Hamburg, Germany, according to the manufacturer's protocol (53). Insulin resistance was calculated using the HOMA-IR (Mass Units) formula, as described previously (54). TNF-alpha was determined by the ELISA technique using PicoKine Kit ABIN411361 from Anticorps SA, Schloss-Rahe-Str15, 52072 Aachen, Germany. Adiponectin was also determined by Elisa, according to the manufacturer's protocol, with Invitrogen Kit (Number; KHP0041) Thermo Fisher Scientific, MA USA. Insulin, TNF-alpha, and adiponectin wavelengths have been determined using Smart Reader ELISA plate reader (Number; 960), Accuris, France.

### Statistical Analysis

Stata 15 and R 3.2.0 softwares were used for statistical analysis of the data. The normality of the distribution of the variables was established using the Shapiro–Wilk test. The χ^2^ test was used to compare the sociodemographic data between exposed and non-exposed participants. ANOVA tests and *post hoc* tests were performed to analyse all the biochemical parameters between exposed and non-exposed groups and also between age groups. For the non-parametric variables, Kruskal–Wallis one-way analysis of variance was preformed. Pearson correlation test between pancreas function test and cholinergic enzyme was also performed. A logistical binominial regression model was used to establish a relationship between the explained variables (exposure) and the explanatory variables (Biochemical Parameters). Odds ratios (ORs) with 95% confidence intervals (95% CIs) were calculated. The differences were considered statistically significant for *p*-value < 0.05.

## Results

The Shapiro-Wilk normality test (Table 1) showed that all the variables used in the study, except for triglycerides, follow normal distribution.

**Table 1:** Normality values of the Shapiro-Wilk Normality test.

**Variable**	**V**	**z**	**Prob>z**
Lipases (UI/L)	126.866	−1.099	0.86416
Amylase (UI/L)	83.170	−1.065	0.85651
Insulin (μUI/mL)	131.647	−0.464	0.67875
Adiponectin (μg/mL)	114.031	−0.653	0.8671
Glycemia (mg/dL)	201.577	−1.103	0.86493
HOMA-IR	268.797	0.281	0.38952
TG (g/L)	52.594	10.532	0.00000
TNF-α (pg/mL)	135.265	−0.707	0.76008
AChE (mU/μmol Hb)	130.234	−1.036	0.81421
BChE (μmol/L/min)	100.634	−1.040	0.79632
Malaoxon (ng/mL)	140.124	−1.006	0.87314
Parathion (ng/mL)	215.367	0.137	0.74256
Chlorpyrifos (ng/mL)	129.513	−1.041	0.81670

*V, variance-covariance matrix; Z, standard score; Prob>z, p-value of the test*.

Table 2 shows the demographic details of the study populations. According to data, females represented 17.81% (52/292) and 55% (165/300) of Pakistani and Cameroonian OP-exposed subjects, respectively. No statistically significant differences were found between gender, age group (16–60), or tobacco consumption with the different biochemical tested variables. We also noted that there was no statistically significant difference between past medical history of diabetes in the family with biochemical variables in all participants in both countries.

**Table 2:** Socio-demographic parameters of the study participants.

**Groups**	**Cameroon**			**Pakistan**		
	**Exposed % (N)**	**Unexposed % (N)**	***P*-value**	**Exposed % (N)**	**Unexposed % (N)**	***P*-value**
Age frequency						
16–30	22 (66)	33 (66)	0.158	46.6 (136)	33.9 (38)	0.146
31–45	42 (126)	44.5 (89)		32.9 (96)	43.8 (49)	
46–61	36 (108)	22.5 (45)		20.5 (60)	22.3 (25)	
Gender frequency						
Female	55 (165)	53.5 (107)	0.102	17.8 (52)	41.1 (46)	≤0.01
Male	45 (135)	46.5 (93)		82.2 (240)	58.9 (66)	
Level of education frequency						
NP	12.3 (37)	12.5 (25)	≤0.01	13.4 (39)	11.6 (13)	≤0.01
SS	38.7 (116)	40.0 (80)		65.1 (190)	58.9 (66)	
OU	49.0 (147)	47.5 (95)		21.6 (63)	29.5 (33)	

*NP, None/Primary school; SS, Secondary school; OU, O-level and Uniersity; Results are significant when P-value ≤ 0.05*.

**Figure 3:**
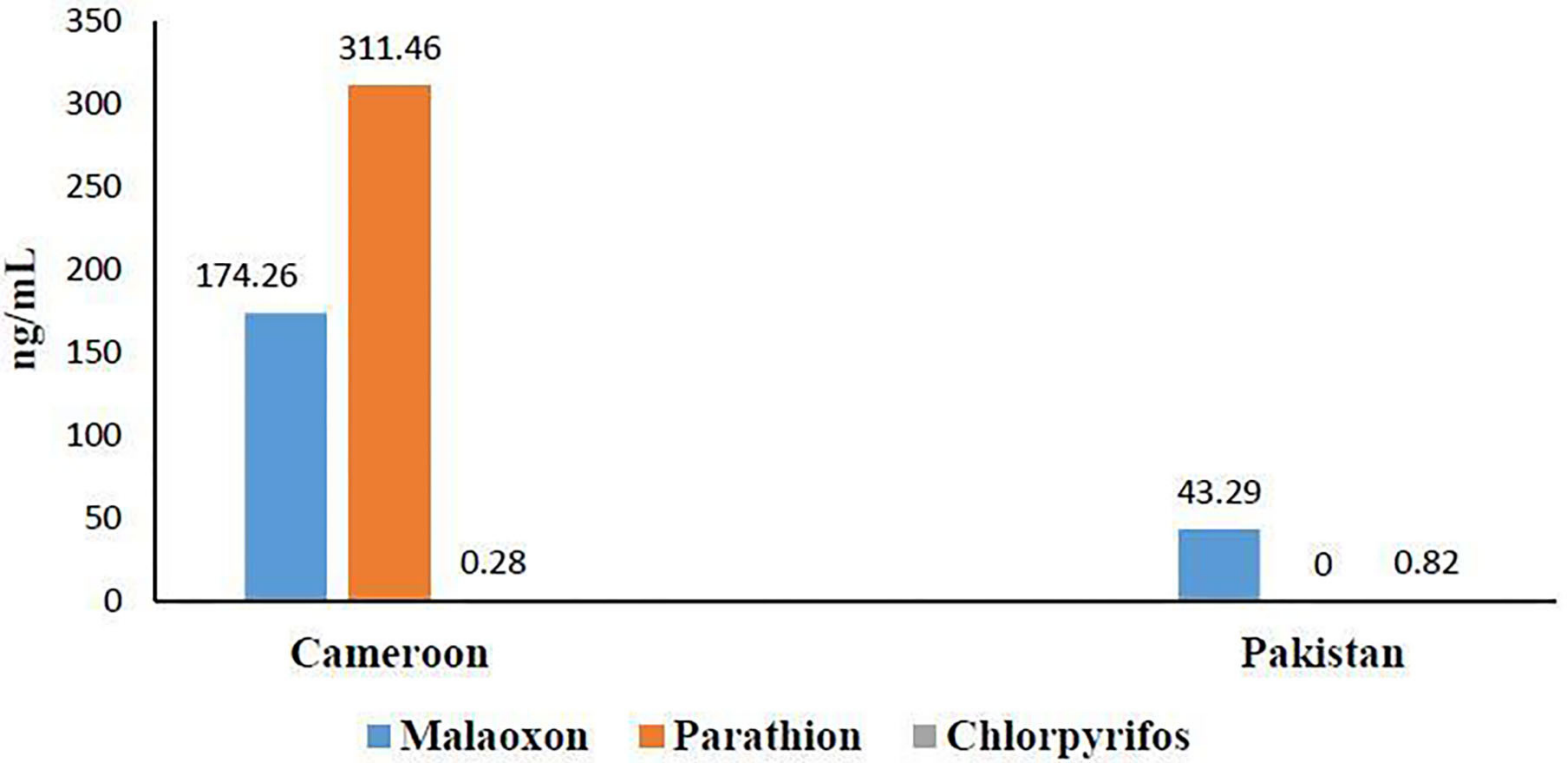
Blood pesticides levels in Cameroonian and Pakistani groups.

OP residues' determination by GC-MS (Figure 3) revealed the presence of three OPs: malathion (174.26 ng/mL ± 101.74), parathion (311.46 ng/mL ± 204.42), and chlorpyrifos (0.28 ng/mL ± 0.25) in Cameroonian OP-exposed individuals. No parathion residues were found in the Pakistani OP-exposed individuals in our detection range. Malathion (43.29 ng/mL ± 37.10) and chlorpyrifos were found to be (0.82 ng/mL ± 0.38) present in the Pakistani group. It is evident from the results that the Cameroonian OP-exposed group has more residues than the Pakistani group.

**Figure 4:**
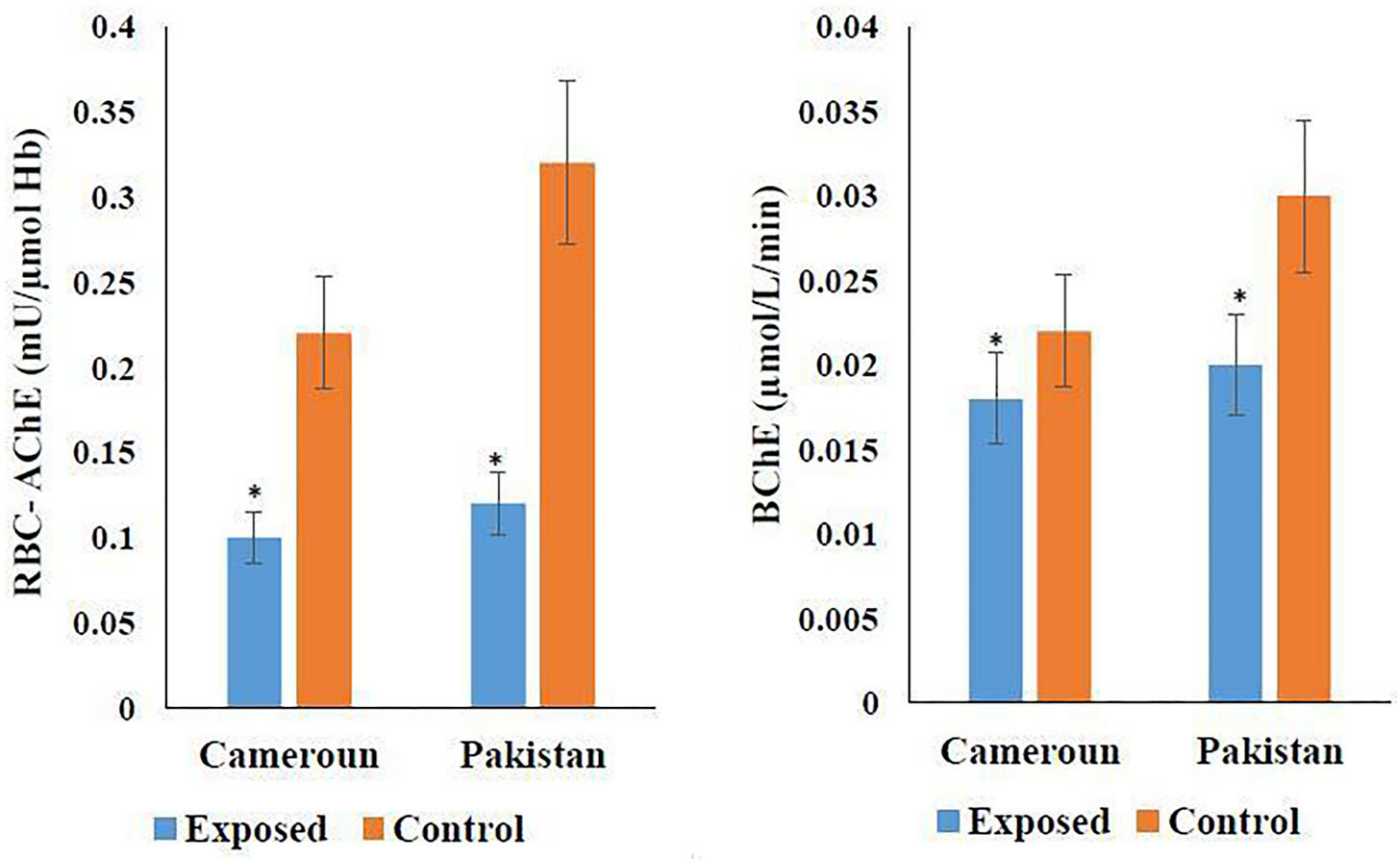
Variation of cholinergic enzymes in Cameroonian and Pakistani groups. ^*^Statistical differnce between exposed and unexposed groups (*P*-value ≤ 0.05).

The cholinergic enzyme activity result is shown in Figure 4. According to Figure 4, 54.54% RBC-AChE was found in Pakistani- and 62.5% in Cameroonian-exposed individuals in comparison to the unexposed healthy group. Reduction in activity is statistically significant. Reduced RBC-AChE indicated that the individuals are exposed to OP and higher depression may be due to the cumulative effect of two/three OPs. BChE activity was also decreased (Figure 4) and statistically significant (*P* ≤ 0.05).

Other investigated biochemical parameters for pancreatic functions (i.e., lipase, amylase, adiponectin, insulin, glucose, triglycerides, HOMA-IR, and TNF-alpha) in exposed subjects compared to unexposed is shown in Table 3. Lipase, insulin, HOMA-IR, and TNF-alpha showed statistically significant increases while amylase and adiponectin in exposed individuals of both countries showed statistically significant decreases (*P* < 0.05).

**Table 3:** Biochemical estimation of lipase, insulin, adiponectin, glycaemia, HOMA-IR, TNFα, and triglyceride in OP-exposed groups from Cameroon and Pakistan.

**Groups**		**Lipase (UI/L) Mean ± SE**	**Amylase (UI/L) Mean ± SE**	**Insulin (μUI/mL) Mean ± SE**	**Adiponectin (μg/mL) Mean ± SE**	**Glycemia (mg/dL) Mean ± SE**	**HOMA-IR—Mean ± SE**	**Triglyceride (g/L) Mean ± SE**	**TNFα (pg/mL) (Mean ± SE)**
Cameroon	Exposed	127.38 ± 3.86^c^^*^	40.58 ± 1.11^b^^*^	17.01 ± 0.73^c^^*^	4.28 ± 1.43^b^^*^	131.05 ± 4.22^*^^b^	6.96 ± 0.03^c^^*^	1.20 ± 0.03 ^*^^b^	21.24 ± 1.12^a^^*^
	Unexposed	91.72 ± 1.75^a^	86.71 ± 0.12^d^	7.27 ± 0.36^a^	9.22 ± 1.17^a^	102.68 ± 1.50^a^	2.05 ± 0.01^a^	1.08 ± 0.01^a^	6.96 ± 0.16^b^
Pakistan	Exposed	107.61 ± 2.91^b^^*^	33.12 ± 0.87^a^ ^*^	13.27 ± 0.37^b^^*^	5.34 ± 1.07^b^^*^	116.53 ± 4.50^a^	4.71 ± 0.02^b^^*^	1.24 ± 0.03^b^	20.94 ± 0.84^a^^*^
	Unexposed	83.48 ± 4.70^a^	71.13 ± 3.68^c^	9.48 ± 0.54^a^	10.76 ± 2.80^a^	99.18 ± 1.97^a^	2.51 ± 0.02^a^	1.23 ± 0.02^b^	5.96 ± 0.11^b^
Overall	Exposed (*N* = 592)	117.49 ± 2.4^*^	36.85 ± 0.99^*^	15.14 ± 0.39^*^	4.81 ± 1.25^*^	123.79 ± 3.08^*^	5.83 ± 0.02^*^	1.22 ± 0.02	21.09 ± 0.98^*^
	Unexposed (*N* = 412)	89.60 ± 3.22	78.92 ± 1.90	8.37 ± 0.28	9.99 ± 1.98	100.93 ± 1.04	2.10 ± 0.01	1.15 ± 0.01	6.44 ± 0.13

* Statistical differnce (P-value ≤0.05);

a,b,c;d *, statistical differnce of post-hoc test (Anova one way) of comparaison Pakistani and Cameroonian populations fot each parameters*.

**Figure 5:**
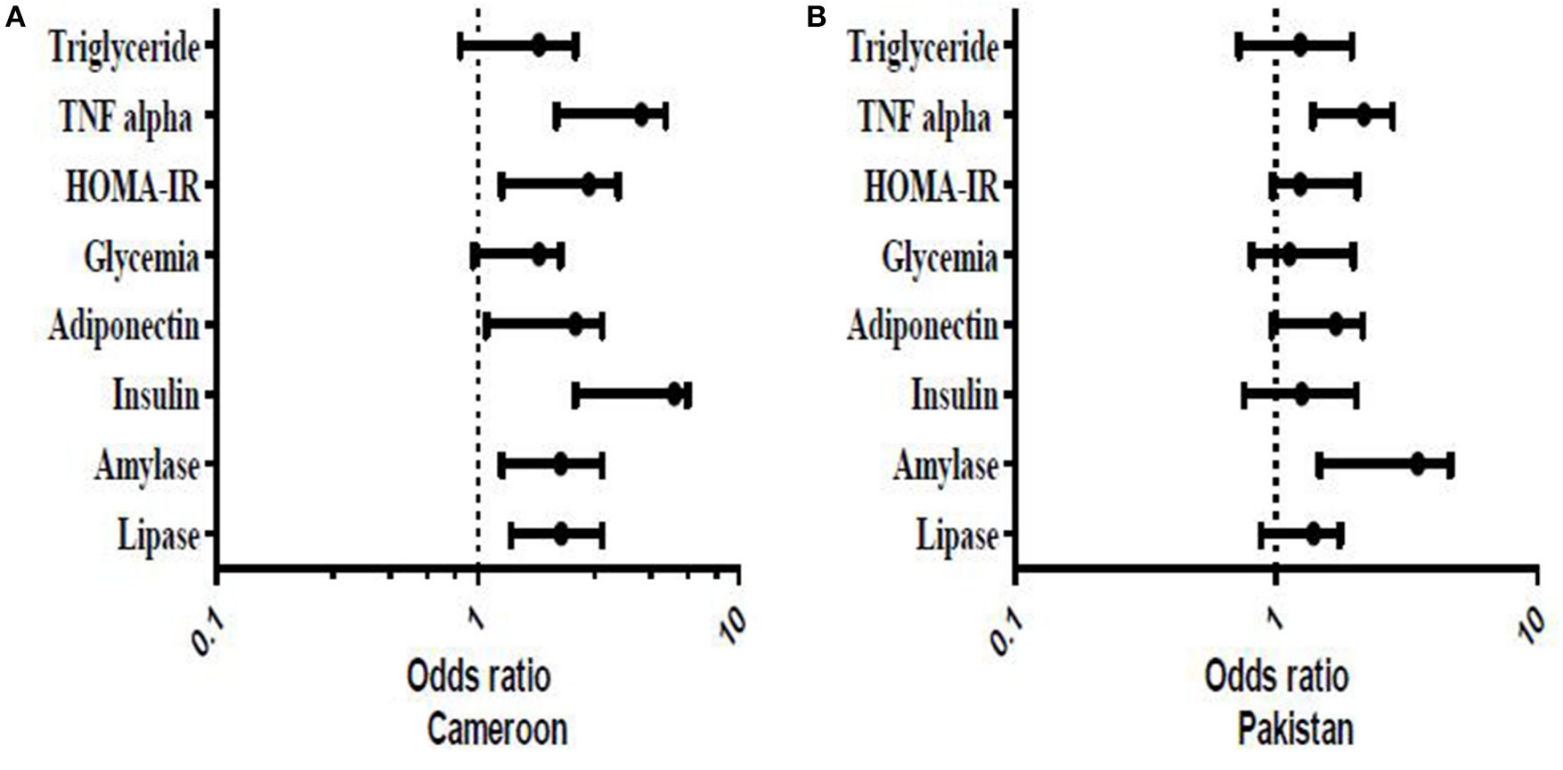
Representation of Binomial Logistic Regression of endocrine and exocrine pancreatic functions tests according of exposure. The model was adjusted (0: exposed individuals; 1: for unexposed individuals). The results were significant if *P* ≤ 0.05.

A representation of the Binomial Logistic regression (BLR) of endocrine and exocrine pancreatic functions test according to exposure (Figure 5) showed the association of OP exposure with a majority of the biochemical tests. For example, the figure shows Cameroonian OP-exposed individuals had 5.680 times more susceptibility to an insulin increase than unexposed subjects (OR:5.680; CI: 2.367–6.378). They also have 2.080 times more chance to have decreased amylase than unexposed subjects (OR:2.080; CI:1.234–2.997) and 2.090 times more chance to have a lipase decrease than unexposed subjects (OR:2.090; CI:1.340–3.010) (Figure 5A). In Pakistani populations, exposed subjects had 3.480 times more susceptibility to decreased amylase than unexposed subjects (OR:3.480; CI:1.467–4.630) (Figure 5B). Logistic regression models for socio-demographic parameters and cofounding factors showed no significance with biochemical parameters at any level.

Pearson correlation results between cholinergic enzymes with other biochemical tests (Table 4A) showed that RBC-AChE and BChE were positively correlated with all the endocrine pancreatic functions test (insulin, blood glucose, and HOMA) and negatively correlated with exocrine pancreatic functions test (lipase and amylase) in both exposed populations. The correlation results between lipase and amylase with other biochemical tests (Table 4B) also showed that insulin, blood glucose, and HOMA-IR values were positively correlated, and adiponectin negatively correlated with lipase and amylase in exposed Pakistani and Cameroonian populations. There was also a correlation between lipase and triglycerides but only in Pakistani OP-exposed individuals. As regards the correlation between lipase with pesticides blood level (Table 4C), we noted a positive correlation between the level of lipase and amylase with blood levels of malathion in Cameroonian OP-exposed individuals, while parathion is correlated and significant only with lipase. The same correlation was also positive (0.1231) and significant among Pakistani OP-exposed individuals with chlorpyrifos. With respect to the biochemical values in the diabetic subjects exposed (Table 5), the mean lipase, amylase, insulin, adiponectin, glycemia, triglyceride, and TNF-alpha levels were 179.02 IU/L, 51.34 IU/L, 21.17 μIU/mL, 4.47 μg/mL, 219.36 mg/dL 1.48 g/L, and 21.24 pg/mL respectively for the Pakistani population and 197.67 IU/L, 48.08 IU/L, 28.42 μIU/mL, 2.34 μg/mL, 203.61 mg/dL, 1.16 g/L, and 21.18 pg/mL for the Cameroonian population. Comparing these results with those of the control diabetic subjects, statistically significant differences are observed (*P* ≤ 0.05) in both countries (Table 5).

**Table 4A:** Correlation of Cholinergic Enzymes with all the biochemical parameters.

**RBC-acetylcholinesterase (RBC-AChE)**										
**Groups**			**Lipase**	**Amylase**	**Insulin**	**Adiponectin**	**Glycaemia**	**HOMA-IR**	**Triglyceride**	**TNF-**α
Cameroon	Exposed	CC *P*-value	−0.637^*^ 0.000	−0.522^*^ 0.000	0.719^*^ 0.000	−0.437^*^ 0.001	0.510^*^ 0.000	0.705^*^ 0.000	−0.081 0.512	0.580^*^ 0.000
	Unexposed	CC *P*-value	−0.146^*^ 0.046	−0.137^*^ 0.041	0.364^*^ 0.000	−0.029 0.608	0.637^*^ 0.000	0.458^*^ 0.000	−0.114 0.200	0.066 0.621
Pakistan	Exposed	CC *P*-value	−0.603^*^ 0.000	−0.430^*^ 0.000	0.640^*^ 0.000	−0.367^*^ 0.001	0.856^*^ 0.000	0.785^*^ 0.000	0.371^*^ 0.000	0.697^*^ 0.000
	Unexposed	CC *P*-value	0.046 0.628	−0.030 0.368	0.417^*^ 0.000	0.100 0.090	0.499^*^ 0.000	0.314^*^ 0.000	−0.074 0.320	0.092 0.605
**Butyrylcholinesterase (BChE)**										
Cameroon	Exposed	CC *P*-value	−0.469^*^ 0.000	−0.457^*^ 0.000	0.418^*^ 0.000	−0.451^*^ 0.000	0.527^*^ 0.000	0.419^*^ 0.000	−0.056 0.567	0.258^*^ 0.001
	Unexposed	CC *P*-value	−0.023 0.127	0.094^*^ 0.497	0.073 0.129	0.074 0.524	0.207^*^ 0.001	0.080 0.127	−0.110 0.100	−0.342^*^ 0.000
Pakistan	Exposed	CC *P*-value	−0.517^*^ 0.000	−0.398^*^ 0.000	0.290^*^ 0.000	−0.248^*^ 0.001	0.710^*^ 0.000	0.625^*^ 0.000	0.233^*^ 0.001	0.044 0.448
	Unexposed	CC *P*-value	0.027 0.713	−0.064 0.573	0.056 0.000	0.081 0.650	0.371^*^ 0.000	0.100 0.092	0.058 0.740	0.073 0.517

* *For significant results (P-value ≤0.05)*;

*CC: correlation coefficient*.

**Table 4B:** Correlation of Lipases and Amylase with Biochemical tests.

**Groups**				**Amylase**	**Insulin**	**Adiponectin**	**Glycaemia**	**HOMA-IR**	**Triglyceride**	**TNF-α**
Cameroon	Exposed	Lipases	CC *P*-value	0.316^*^ 0.000	0.601^*^ 0.000	−0.367^*^ 0.001	0.690^*^ 0.000	0.610^*^ 0.000	−0.047 0.412	0.056 0.326
	Unexposed		CC *P*-value	0.483^*^ 0.000	0.443^*^ 0.000	−0.036 0.527	0.712^*^ 0.000	0.591^*^ 0.000	−0.100 0.156	−0.042 0.549
Pakistan	Exposed		CC *P*-value	0.837^*^ 0.000	0.778^*^ 0.000	−0.412^*^ 0.001	0.942^*^ 0.000	0.864^*^ 0.000	0.263^*^ 0.000	0.053 0.366
	Unexposed		CC *P*-value	0.703^*^ 0.000	0.624^*^ 0.000	0.076 0.570	0.602^*^ 0.000	0.656^*^ 0.000	−0.087 0.358	0.051 0.591
Cameroon	Exposed	Amylase	CC *P*-value		0.199^*^ 0.000	−0.388^*^ 0.001	0.180^*^ 0.001	0.194^*^ 0.000	−0.032 0.575	0.071 0.215
	Unexposed		CC *P*-value		0.255^*^ 0.000	0.094 0.421	0.280^*^ 0.000	0.272^*^ 0.000	−0.115 0.103	−0.142^*^ 0.043
Pakistan	Exposed		CC *P*-value		0.640^*^ 0.000	−0.364^*^ 0.001	0.816^*^ 0.000	0.730^*^ 0.000	0.174^*^ 0.002	0.044 0.448
	Unexposed		CC *P*-value		0.575^*^ 0.000	0.067 0.641	0.399^*^ 0.000	0.533^*^ 0.000	0.010 0.912	0.042 0.653

* *For significant results (P-value ≤0.05)*;

*CC: correlation coefficient*.

**Table 4C:** Correlation of lipases and amylases with pesticides residues.

**Groups**				**Malathion (ng/mL)**	**Parathion (ng/mL)**	**Chlorpyrifos (ng/mL)**
Cameroon	Exposed	Lipases	CC *P*-value	0.709^*^ 0.000	0.416^*^ 0.000	0.005 0.993
	Unexposed		CC *P*-value	-	-	-
Pakistan	Exposed		CC *P*-value	0.068 0.246	-	0.123^*^ 0.035
	Unexposed		CC *P*-value	-	-	-
Cameroon	Exposed	Amylase	CC *P*-value	0.110^*^ 0.056	−0.048 0.403	−0.038 0.503
	Unexposed		CC *P*-value	-	-	-
Pakistan	Exposed		CC *P*-value	0.053 0.363	-	0.133^*^ 0.042
	Unexposed		CC *P*-value	-	-	-

* *For significant results (P-value ≤0.05)*;

*CC: correlation coefficient*.

**Table 5:** Variation of Biochemical tests according to Type 2 diabetes.

**Biochemicals**	**Cameroon**				**Pakistan**			
	**1: Diabetic-Exposed *N* = 102**	**2: Diabetic only *N* = 21**	**3: Exposed without Diabetes *N* = 198**	***P*-value**	**1: Diabetic-Exposed *N* = 70**	**2: Diabetic only *N* = 18**	**3: Exposed without diabetes *N* = 221**	***P*-value**
Lipase	197.67 ± 65.73a	129.38 ± 48.25b	91.19 ± 25.88c	0.001=1&2 0.001=1;2&3	179.02 ± 47.9a	164 ± 60.01a	83.37 ± 14.75c	≥0.05: 1&2 0.001=1;2&3
Amylase	48.08 ± 26.04	102.95 ± 36.22	36.72 ± 13.15	≥0.05: 1&3 0.001=1;2&3	51.34 ± 15.66	112.50 ± 47.08	26.94 ± 7.99	0.001=1;2&3
Insulin	28.42 ± 14.18a	18.00 ± 4.99b	11.16 ± 5.85c	0.001=1&2 0.001=1;2&3	21.17 ± 7.01a	18.59 ± 6.71a	10.58 ± 2.79b	≥0.05:1&2 0.001=1;2&3
Adiponectin	2.34 ± 1.49b	7.16 ± 0.67a	6.22 ± 1.37a	0.001=1&2 0.05=1;2&3	4.47 ± 0.96b	6.98. ± 1.43a	6.21 ± 1.18a	≥0.05: 1;2&3
Glycemia	203.61 ± 85.80a	143.90 ± 33.37b	93.67 ± 12.79c	0.001=1&2 0.001=1;2&3	219.36 ± 92.30	139 ± 16.92b	82.17 ± 13.73c	0.001=1&2 0.001=1;2&3
TG	1.16 ± 0.55a	1.08 ± 0.14b	1.21 ± 0.52a	0.01=1&2 ≥0.05:1;2&3	1.48 ± 0.53a	1.26 ± 0.19a	1.15 ± 0.41b	≥0.05: 1&2 0.001=1;2&3
TNF alpha	21.18 ± 10.81	7.03 ± 2.16	21.27 ± 8.04	≥0.05: 1&3 0.001=1;2&3	21.24 ± 13.90a	6.17 ± 1.15b	20.84 ± 14.42a	≥0.05: 1&3 0.001=1;2&3

*Statistical differnce (P-value ≤ 0.05); a,b,c;d = statistical differnce of post-hoc test (Anova one way) of comparaison diabetic exposed, diabetic only and exposed without diabete groups in Pakistani and Cameroonian populations fot each parameters. TG, tryglyceride; TNF, tumor necrosis factor.

## Discussion

Organophosphorus compounds account for more than 80% of the total insecticides/pesticides used across the world. Globally 150,000 people die each year due to pesticide poisoning (56). Intoxication and maladies due to acute and chronic exposure in populations is regularly reported in literature (57–61). A mixture of OPs, even at low non-lethal doses, may pose an enhanced threat because of the possibility of a synergistic effect between two OPs (45, 62). Pancreatic intoxication and dysregulation is among the many results from OP toxic manifestation (27, 62, 63) but are mainly reported for acute OP toxicity in literature. Our results showed the presence of three OPs in Cameroonian and two OPs in Pakistani exposed groups. Marked inhibition (54.54% and 62.50%) of RBC-AChE was found in both population groups, which is not only indicative of the exposure of OP but may also represent a synergistic effect. The results showed that OP exposure resulted in a statistically significant elevation of insulin, blood glucose, HOMA-IR, lipase, TNFα, and triglycerides in OP-exposed individuals compared to non-exposed Cameroon and Pakistani participants. Contrary to the Cameroonian exposed individuals, there was no statistically significant difference in the levels of triglycerides between exposed and unexposed people in the Pakistani population. A statistically significant decrease of amylase and adiponectin in OP-exposed individuals compared to unexposed participants in each country was also found. OP pesticide exposure affecting diabetes in humans and animal models has been documented, but the results have been contradictory. Notably, an increase in blood glucose, insulin, HOMA-IR, triglycerides, and lipase in subjects exposed to organophosphorus pesticides have been reported by a number of studies (64–70). Other studies have shown a decrease in the amount of these metabolites after exposure to OP (71, 72). There are also studies that have reported no effect on certain metabolites, such as blood glucose, upon exposure to OP (73).

No significant statistical differences between gender and biochemical parameters investigated in the context of exposure to OP was found. However, other studies have investigated different pesticides levels in females and males (74–76). According to the literature reviewed, we could not identify any study that explored the relationship between these biochemical parameters assessed in the present study and gender in the context of OP exposure. A strong correlation was observed for insulin, blood glucose, and HOMA-IR with amylase and lipase in both Pakistani and Cameroonian OP-exposed individuals. This could be due to the exocrine and endocrine functions of the pancreas, as both are dependent on each other (33). This could be seen in diabetics where impaired endocrine function results in acute and chronic pancreatitis, which impaired the exocrine role (11, 34, 76, 77). The correlation between exocrine secretions' amylase and lipase, with the usual diagnostic parameters of diabetes, suggest that exocrine secretions could not only be a diagnostic marker but also an index for other disease-related physiological complications. A correlation between lipase levels and triglycerides were found to be correlated in the Pakistani group. This could be due to obesity, metabolic disorders, and low-grade inflammation in diabetics (78, 79). A positive correlation was observed between serum/plasma lipase and pesticides. Parathion and malathion were positively correlated in Cameroonian OP-exposed individuals. There was a positive correlation with the rate of chlorpyrifos in Pakistan's OP group. This positive correlation could be due to two mechanisms: the direct effects of OP pesticides on certain target organs, such as the pancreas, resulting in pathologies such as pancreatitis; or the other indirectly related to the pathophysiology caused by acute high-dose exposure or accumulation of pesticides in our body resulting in diseases such as obesity, diabetes, and cardiovascular dysfunction (54, 80–84). In our study, there is considerable variation in the biochemical tested parameters as a function of diabetic status and pesticide exposure of subjects in both populations (Cameroon and Pakistan). The parameters lipase, insulin, and glycemia are much higher in OP-exposed diabetic subjects; this suggests that exposure to organophosphorus pesticide form a gravity factor for diabetic's conditions. Our results are in concordance with earlier studies (85, 86). The Cameroonian population is much more sensitive to this variation compared to the Pakistani population; for example, the lipase value is 197.67 in OP-exposed diabetic subjects in the Cameroonian population against 179.02 in the Pakistani population. These differences could be caused by several factors, such as the level of difference in pesticide tolerance of individuals in two countries, genetic differences, or the level of exposure to pesticides, in addition to genetic predispositions of pesticides hydrolyzing enzymes like paraoxonases in two distinct populations. It is noteworthy from the results that two distinctly different groups respond variably to three OPs. In addition, three different OPs showed different toxicity levels. It is obvious that malathion, parathion, and chlorpyrifos are structurally different OPs and, despite having the same mechanism of toxic action, prove that structurally different OPs show differences in toxicity.

Using the model of logistic regression, it was observed that the subjects exposed to OP pesticides were twice as likely to have higher than normal serum amylase in comparison with unexposed subjects in the Cameroonian population. The same observation was made for insulin and triglyceride. In the Pakistani population, the risk of having higher than normal values for the various metabolic parameters investigated were 1.39 for amylase and 3.48 for lipase in exposed subjects compared to the unexposed ones. These observations support the risk of occurrence of serious metabolic pathologies in the population exposed to OP pesticides. Although age and smoking are risk factors for cardio-metabolic diseases (87), no statistically significant differences between biochemical substances, age, and smoking were found in subjects of both countries.

## Conclusion

Organophosphates residues and marked depression of RBC-AChE and BChE in both populations were evident. Cameroonian groups were more sensitive to OP and showed a notable decrease of cholinergic enzymes. Data analysis and logistic regression showed higher risks of diabesity and pancreatic dysregulation among OP-exposed groups, leading to altered endocrine and exocrine secretions. In addition, the results suggest that lipase and amylase may be a potential alternate marker for OP poisoning and diabetes under OP exposure. Further occupational and environmental exposure studies with mixture of pesticides and their impact on public health in the region is suggested.

## Limitations

There are certain limitations to the study which are noteworthy to mention here. For instance, biomarkers from urinary metabolites could not be carried out because of the non-availability of substantial samples. In addition, it is a cross-sectional study, but all the parameters may not have been employed due to some unavoidable reasons. Similarly, OPs supposed to be intensively used in the chosen regions in Pakistan and Cameroon and the presence of other potential confounders, such as exposure to other organic pollutants and heavy metals such as Arsenic or Lead, have not been considered. The study also concluded that OP can cause pancreatic dysregulation and diabetes in two population countries, but the authors understand that lifestyle factors may have some influence on diabetes, which is missed in the present study. However, it is noteworthy that OP induced pancreatitis, diabesity, and other metabolic disorders have been documented in the literature.

## Data Availability Statement

The datasets generated for this study are available on request to the corresponding author.

## Ethics Statement

The studies involving human participants were reviewed and approved by COMSATS University Islamabad, Pakistan (CIIT/BIO/ERB/19/90) and Cameroon National Ethical Committee (#488/CE/CNERSH). The patients/participants provided their written informed consent to participate in this study.

## Author Contributions

ML conceived the study, collected samples, performed experiments, and wrote the manuscript. SR performed statistical analysis and critically reviewed the work. NJ worked as a supervisor in Cameroon and critically reviewed the manuscript. FA substantially arranged and reviewed the data. RH significantly reviewed and revised the manuscript. SB supported interpretations of data and the revision of the manuscript. SN and ML conceived the study, supervised, and revised the manuscript to its final draft. All authors contributed to the article and approved the submitted version.

## Conflict of Interest

The authors declare that the research was conducted in the absence of any commercial or financial relationships that could be construed as a potential conflict of interest.
